# A Proteomic and Cellular Analysis of Uropods in the Pathogen *Entamoeba histolytica*

**DOI:** 10.1371/journal.pntd.0001002

**Published:** 2011-04-05

**Authors:** Jacques Marquay Markiewicz, Sylvie Syan, Chung-Chau Hon, Christian Weber, Daniela Faust, Nancy Guillen

**Affiliations:** 1 Institut Pasteur, Unité Biologie Cellulaire du Parasitisme, Paris, France; 2 INSERM U786, Paris, France; Bose Institute, India

## Abstract

Exposure of *Entamoeba histolytica* to specific ligands induces cell polarization via the activation of signalling pathways and cytoskeletal elements. The process leads to formation of a protruding pseudopod at the front of the cell and a retracting uropod at the rear. In the present study, we show that the uropod forms during the exposure of trophozoites to serum isolated from humans suffering of amoebiasis. To investigate uropod assembly, we used LC-MS/MS technology to identify protein components in isolated uropod fractions. The galactose/N-acetylgalactosamine lectin, the immunodominant antigen M17 (which is specifically recognized by serum from amoeba-infected persons) and a few other cells adhesion-related molecules were primarily involved. Actin-rich cytoskeleton components, GTPases from the Rac and Rab families, filamin, α-actinin and a newly identified ezrin-moesin-radixin protein were the main factors found to potentially interact with capped receptors. A set of specific cysteine proteases and a serine protease were enriched in isolated uropod fractions. However, biological assays indicated that cysteine proteases are not involved in uropod formation in *E. histolytica*, a fact in contrast to the situation in human motile immune cells. The surface proteins identified here are testable biomarkers which may be either recognized by the immune system and/or released into the circulation during amoebiasis.

## Author Summary

Uropods are membrane folds formed at the rear of moving cells, e.g. lymphocytes during immune responses and the amoebic parasite *Entamoeba histolytica* during amoebiasis. Previous studies showed some surface receptors of *E. histolytica*, e.g. the Gal/GalNAc lectin, which is involved in adhesion, undergo capping and accumulate at the uropod, and these processes are driven by the activities of the actin-rich cytoskeleton. These uropods are then discarded to the extracellular medium, suggesting the components of uropods may induce anti-amoebic responses from the host. In this study, we showed that the serum from patients infected with *E. histolytica*, but not serum from healthy individuals, is able to induce uropod formation. To characterize the proteome of these induced uropods, we performed a proteomic analysis of the discarded complexes. In addition to the presence of several proteases and novel cytoskeleton factors, our proteomic results highlight the presence of important surface components including the Gal/GalNAc lectin, calreticulin, several adhesion molecules and the immunodominant antigen M17. Finally, we derived two important conclusions from further cellular analyses. Firstly, cysteine proteases are not involved in uropod formation in *E. histolytica*. Secondly, M17 was confirmed to be recruited at the uropods induced by serum from infected patients.

## Introduction

The acquisition of cell polarity is a crucial requirement for motility in a variety of cells, including activated leukocytes and fast-moving amoebae. Cell polarization is defined by the presence of an anterioposterior cell axis and two functionally and morphologically distinct poles: the leading edge, which guides the cell's directional movements, and the trailing edge (i.e. the uropod), which accumulates adhesion molecules. Following surface receptor activation and subsequent patching and capping, uropods form concomitantly with a retrograde flow of the cortical actomyosin cytoskeleton. It has been suggested that these dynamic properties are closely related to how cells move [1], [2]. However, an important body of evidence indicates that uropods are essential for other relevant cell functions, such as cell-cell communication and cell adhesion. Uropods are found in neutrophils, monocytes, natural killer cells and amoebae and appear to have an important role in immune-related interactions [3]. For instance, adhesion molecules are recruited into cellular uropods following exposure to chemokines. This process constitutes an important step in the mechanism responsible for the recruitment of leukocytes to the inflammation site. Although these phenomena are involved in immune responses during inflammation (in the case of leukocytes) or infection (in the case of amoebic parasites), the interplay between uropod formation and surface receptor capping is still poorly characterized.

Human amoebiasis is a persistent, infectious disease whose symptoms vary from amoebic colitis with destruction of the intestinal epithelium and severe dysentery to extra-intestinal abscesses particularly in the liver [4], [5]. In amoebiasis, the parasite *Entamoeba histolytica* employs a range of diverse strategies for immune evasion. The most distinctive strategy is surface receptor capping, in which surface targets for host immune components are translocated towards the uropod and then released into the culture medium [6], [7]. This membrane shedding also enables *E. histolytica* to discard bound, harmful substances such as anti-amoeba antibodies and complement. Surface receptors circulate between the cell surface and the intracellular compartment via internalization in active endocytic processes. The residence time of these surface receptors in the endocytic compartment depends on the receptors' functions. The fact that uropods are discarded from the cells (thus reducing the extentn of endocytosis) suggests that (i) the isolated fraction concentrates various molecules to the plasma membrane and (ii) the excreted molecules are likely to have a relevant effect on the establishment of amoebiasis. Therefore it is essential to identify the major components of discarded fractions to understand the mechanism of uropod formation.

During invasive amoebiasis, *E. histolytica* attaches to its target cell via the galactose/N-acetylgalactosamine lectin (Gal/GalNAc) and performs contact-dependent cell killing [8]. Although the main target cell-binding protein Gal/GalNAc is not exclusively expressed at the cell surface, it is an immunodominant molecule which can induce IgA antibody secretion in amoebiasis patients [9]. The Gal/GalNAc lectin is composed of two subunits: a 170 kDa heavy chain (HgL) with a transmembrane domain and a cytoplasmic tail with motifs sheared with the signalling molecule β2 integrin (an integrin receptor subunit involved in cell-cell adhesion) [10], and a 30/35 kDa light chain (LgL). The LgL subunit is attached to the membrane by a GPI anchor and to the heavy chain via disulfide bonds. The complex is associated with the 120-kDa intermediate subunit (IgL) [11], [12], which also contains a GPI anchor. When *E. histolytica* is incubated in the presence of lectins such as concanavalin A (Con A, which has been widely used to investigate receptor capping), the Gal/GalNAc lectin accumulates at the uropod [13], [14], [15]. Remarkably, blocking out-to-in signalling by using a dominant negative strategy against the HgL subunit [10], [15] leads to a reduction in parasite adhesion to cells and in Gal/GalNAc lectin clustering of receptors by Con A. The HgL dominant negative parasites are unable to move and thus impact pathogenesis since these do not produce effective liver infection in the hamster model of hepatic amoebiasis [16], [17]. However, these amoeba are still able to invade the human colon effectively in an experimental model of intestinal amoebiasis [18]. Interaction between the HgL carboxyl-terminal domain and the amoebic cytoskeleton (via actin-binding proteins such as α-actinin) [19] is a key step in this signalling pathway and determines the tissue specificity of Gal/GalNAc lectin. Recently, the light chains have also been found to be important for Gal/GalNAc lectin capping activity, since the absence of LgL subunits 1 to 3 affects the parasites' ability to cap and translocate the Gal/GalNAc lectin to the uropod region [20]. Insight into the capping process's mechanism has also been gained recently: a serine protease from the rhomboid family concentrates in the vicinity of the uropod and cleaves the Gal/GalNAc HgL subunit *in vitro* [21]. These findings highlight the potential role of a large number of amoebic proteases in surface receptor capping and uropod formation. Functional links between proteinases and uropod formation have also been observed in other eukaryotic cells. For instance, leukocyte migration is promoted by the activity of cathepsin X, a cysteine peptidase localized at the uropod and which modulates the interaction between β2 integrin and the actin-rich cytoskeleton [22], [23]. In addition to the Gal/GalNAc lectin, calreticulin (CRT) was found to be another antigen localized in the uropod in addition to its localization in the endoplasmic reticulum [24]. CRT has an important role in a variety of cellular processes, including Calcium signalling and protein folding. The fact that CRT is an immunodominant antigen during hepatic amoebiasis [25] suggests that it may be involved in the onset of inflammation and the immune response.

Receptor capping at the amoebic surface and then extrusion of uropod fractions both require active remodelling of the actomyosin cytoskeleton [13], [26]. These cytoskeleton functions are regulated by a panel of important proteins, including the small GTPases RacG [27] and RacA [28], their corresponding GTP exchange factors [29], [30], [31], the PAK kinases [32], [33] and the actin-filament cross-linker Filamin A (previously referred to as ABP120) [34]. Blocking myosin II inhibits surface receptor capping and, as a result, trophozoites are unable to invade living tissues [15].

To gain insight into the molecular composition of uropods, we performed a high-throughput LC-MS/MS proteomic analysis of the uropod-extruded fraction following incubation of *E. histolytica* with Con A. Our results confirmed the expected presence of the Gal/GalNAc lectin and CRT. In addition, our results also suggest the presence of immunodominant variable surface antigen M17 [35], a number of proteins involved in multiple drug resistance [36] , a set of specific ATPases, a number of small GTPases, cysteine proteases, at the uropod enriched fractions. Given the potential roles of immunodominant M17 antigen and cysteine proteases in the pathogenesis of amoebiasis, we verified the enrichment of M17 at uropod and investigated the potential roles cysteine proteases in uropod formation, using cell biology approaches. To the best of our knowledge, this is the first report on the uropod proteome in any cell. The *E. histolytica* surface proteins identified in this study may provide new insights into the biology of the parasite. Indeed, the uropod components appear to be testable biomarkers which may be either recognized by the immune system and/or released into the blood. The molecular and cellular analysis of uropod extruded fractions thus opens up opportunities for better understanding the mechanism of amoebic infection.

## Methods

### Parasite culture and cysteine protease inhibition

The pathogenic *Entamoeba histolytica* (wild type, HM1: IMSS strain) was cultured axenically in TYI-S-33 medium [37] at 37°C. For protease inhibition tests, 1.8×10^5^ trophozoites in 1,5 ml of TY-S-33 medium were incubated for 3 h at 37°C in the presence of (2S, 3S)-trans-Epoxysuccinyl-L-leucylamido-3-methylbutane (E-64c, Sigma) or (2S, 3S)-trans-Epoxysuccinyl-L-leucylamido-3-methylbutane ethyl ester (E-64d, Sigma) [38]. 100 µM of both E64 were used, at this concentration the enzymatic activity of cysteine proteases is inhibited by 95% as measured by the degradation of the synthetic substrate Z-RR-AMC (Sigma) (data not shown). Then the parasites were washed in PBS twice, resuspended in 1 ml of PBS and incubated in the presence of Con A (20 µg/ml) as described below.

### Imaging of live *E. histolytica*

For video microscopy, the parasites (10^5^ per ml) in PBS were seeded on glass bottom culture dishes (MatTeck) and incubated at 37°C in the presence of 5 µg/ml of fluorescent Con A (Alexa fluor 488, Molecular Probes). Live parasites undergoing capping were imaged using a confocal microscope (40× objective) with a Nipkow disk device (Perkin Elmer). Images (10 per second) were processed with ImageJ software (http://rsb.info.nih.gov/ij).

### Induction of receptor capping and purification of uropod extruded fractions

Amoeba trophozoites (5×10^8^) were incubated in the presence of Con A (20 µg/ml) (grade VI; Sigma) at 4°C for 1 h. To induce cap formation and release, the cells were moved to 37°C for 10 min and then harvested. The protein fractions were extracted and treated in accordance with previously published methods [13]. Briefly, trophozoites and cellular debris were eliminated by two successive centrifugations at 300× *g* for 5 min. Caps were pelleted at 30,000× *g* for 30 min at 4°C. The final pellet was resuspended and washed twice in 100 ml of PBS containing 1 M α-methyl-D-mannopyranoside and protease inhibitors (2 mM AEBSF, 1 mM NEM, and 2 mM PHMB). Lipids were removed by washing the pellet in methanol (600 µl), chloroform (150 µl), water (450 µl) and centrifugation at 1000 g for 5 min. The aqueous phase was treated with methanol (450 µl), centrifuged at 1000 g for 5 min and the pellet was dried. The protein fraction and crude extract from growing trophozoites (10 µg) were analyzed by western blot with an anti-Gal/GalNAc lectin antibody prepared in our laboratory [14] against the tail domain of HGL subunit (dilution 1∶400) and with an anti-ConA antibody (Sigma) diluted 1∶500. Detection was performed with a secondary anti-rabbit antibody and enhanced chemoluminescence.

### Protein analysis by liquid chromatography and tandem mass spectrometry (LC-MS/MS)

Two independent experiments were performed. The dried protein pellet (100 µg, obtained from 10^8^ cells) was dissolved in 20 µl of 1% SDS and then slowly diluted with 50 mM ammonium bicarbonate to a final concentration of 0.1% SDS. The sample was reduced with DTT and alkylated with iodoacetamide before digestion with 1 µg of modified trypsin (Promega) for 24 hrs at RT. A second 1 µg of trypsin was added and digestion was allowed to proceed for an additional 24 hrs. The sample was then desalted and ion-exchanged before concentration. Around 30% of the digest was introduced into the mass spectrometer for analysis. Two runs (technical replicates) were performed using slightly different instrument data acquisition parameters, so that as many different proteins as possible could be identified. The full LC-MS/MS procedure was performed by the Biomolecular Research Facility at Virginia University (1300 Jefferson Park Avenue, Jordan Hall, Room 1101, Charlottesville, VA 22908, USSA, tel. +1 434 924-2356). The LC-MS system consisted of a Finnigan LTQ-FT ion trap - ion cyclotron resonance mass spectrometer system with a Protana nanospray ion source, interfaced to a self-packed 8 cm×75 mm id Phenomenex Jupiter 10 mm C18 reversed-phase capillary column. 0.5–10 µl volumes of the extract were injected and the peptides were eluted from the column by an acetonitrile/0.1 M acetic acid gradient at a flow rate of 0.25 µl/min. The nanospray ion source was operated at 2.8 kV. The digest was analyzed using the instrument's double-play capability by acquiring (i) full scan mass spectra to determine peptide molecular weights and (ii) product ion spectra to determine the amino acid sequence in sequential scans. This mode of analysis produces approximately 10,000 collisionally-activated dissociation (CAD) spectra of ions ranging in abundance over several orders of magnitude. Not all CAD spectra were derived from peptides. The data were processed using Sequest in the Thermo Electron Bioworks program ver 3.3.1 (instrument software Xcalibur 2.0) against the *E. histolytica* proteome downloaded from NCBI (genome version 2005). Parent mass tolerance of 8 ppm, fragment ion tolerance of 0.8 Da. The Xcorr scores adopted the following thresholds: +1>1.8, +2>2.2, +3>2.7, +4(and higher)>3.5. The search included CAM Cys as a static modification and Ox Met as a differential modification. Then Scaffold version 3_00_03 computer program was then used to analyse the data (569 proteins were identified with 0,2% of FDR). Then identity of proteins was further confirmed in the *E. histolytica* reannotated genome (Pathema, data version 5.0). The complete data set was deposited to Tranche database (https://proteomecommons.org/tranche/). The accession number of the dataset, which is called “hash”, is: C94k72mNyTtPf6PDjuFbpsRJUviEokUNGt6joLkgwIJuSNl5SYz/iruupzJPKc1CZabar3up98e2syGVm/g75qEjnVQAAAAAAAAB3g =  = .

### Immunofluorescence staining and confocal microscopy

Trophozoites (2×10^6^/ml) were incubated in PBS containing fluorescent Con A (5 µg/ml) and non-fluorescent Con A (10 µg/ml) or human serum (40 µl/ml) for 5 min at 37°C. Naive human serum (n = 3) was obtained through the Pasteur Institute's DIAGMICOLL project (registration n° RBM #816) and sera from patients (n = 2) were a gift from Dr M. C. Rigothier (Faculty of Pharmacy Châtenay-Malabry, France). Three experiments were conducted. The parasites were fixed in 3.7% formaldehyde for 30 min and incubated in 50 mM NH_4_Cl/PBS for 30 min. In some cases, cells were permeabilized by adding 0.1% Triton ×100 for 1 min and the samples were then blocked in 1% BSA/PBS for 30 min at 37°C. Preparations were then incubated with the following antibodies (diluted 1∶100) raised in rabbits: anti-M17 (raised against the peptides GTKPKEWTMKYTKYP and ENNFESKYSIKRDST in this work), and anti CP-A5 or anti-CP-A1-A2 [39], a kind gift of Dr Tomoyoshi Nozaki (National Institute of Infectious Diseases, Japan). The anti-human or anti-rabbit secondary antibodies coupled to Alexa 488 or Cy3 (Molecular Probes) were added at a dilution of 1∶200 for 30 min at 37°C. Amoeba were examined by confocal microscopy (microscope LSM510, Zeiss). When necessary the number of uropods was determined in the total fraction of counted parasites summed from the three experiments.

### Substrate gel electrophoresis

1 µg of uropod proteins was migrated on 10% SDS-polyacrylamide gel co-polymerized with 0.1% gelatine (w/v). After removal of SDS by shaking the gel in 2.5% Triton X-100 for 30 min, and subsequent incubation of the gels overnight at 37°C in 0.1 m sodium phosphate buffer pH 6.5, containing 2,5 mM dithiothreitol, gelatinase activity was detected as a clear band in the Coomassie Brilliant Blue-stained gels.

## Results

### Uropod formation and release from *E. histolytica*

**Figure 1:**
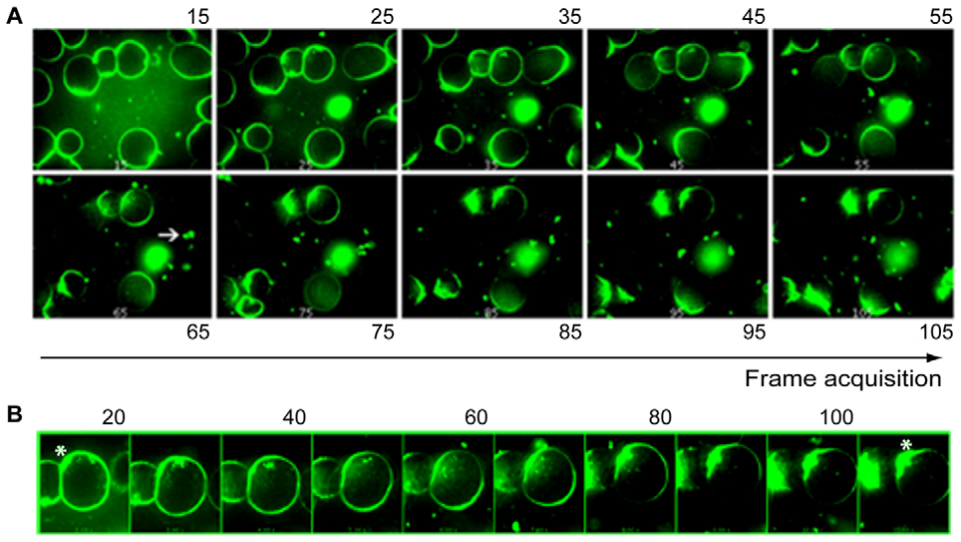
Spatiotemporal analysis of the redistribution of Con A associated with the surface of *E. histolytica*. Trophozoites (of 20–30 µm size) were incubated under the microscope at 37°C and fluorescent Con A was added at the starting time point. *In vivo* imaging was performed and the uropod formation process was detected by frames (indicated by numbers) recorded in a confocal microscope with a Nipkow disk device. A: the micrograph represents 100 images recorded as 10 images every second. Note the polarisation of fluorescent Con A over time and the increase in the extrusion of particles into the medium (white arrow). B: Enlarged frames from a chosen cell are presented, with the white star marking the end of the trophozoite at which the uropod is formed. The entire sequence lasted 11 seconds. See Video S1 for visualisation of details in real time.

To determine the kinetics of uropod formation and release, we imaged live parasites incubated with fluorescent Con A (FITC-Con A). The process was observed by rapid acquisition in confocal laser microscopy using a Nipkow disk device (Video S1 and Figure 1). Initially, Con A bound uniformly to the cell surface and indicated a symmetrical receptor distribution. Activation of receptor capping induced changes in the cell shape and probably accounted for the asymmetrical distribution of the FITC-Con A ligand; the latter was absent from the front of the cell but was concentrated in the uropod (Figure 1A). Internalization of ligand-receptor complexes was observed in some cells as intracellular fluorescent spots, indicating the presence of an active endocytic process during receptor capping. The process of uropod formation and release occurred rapidly (in 5 to 10 seconds) (Figure 1B). The extruded fractions accumulated in the medium and were observed as extracellular fluorescent agglomerates.

**Figure 2:**
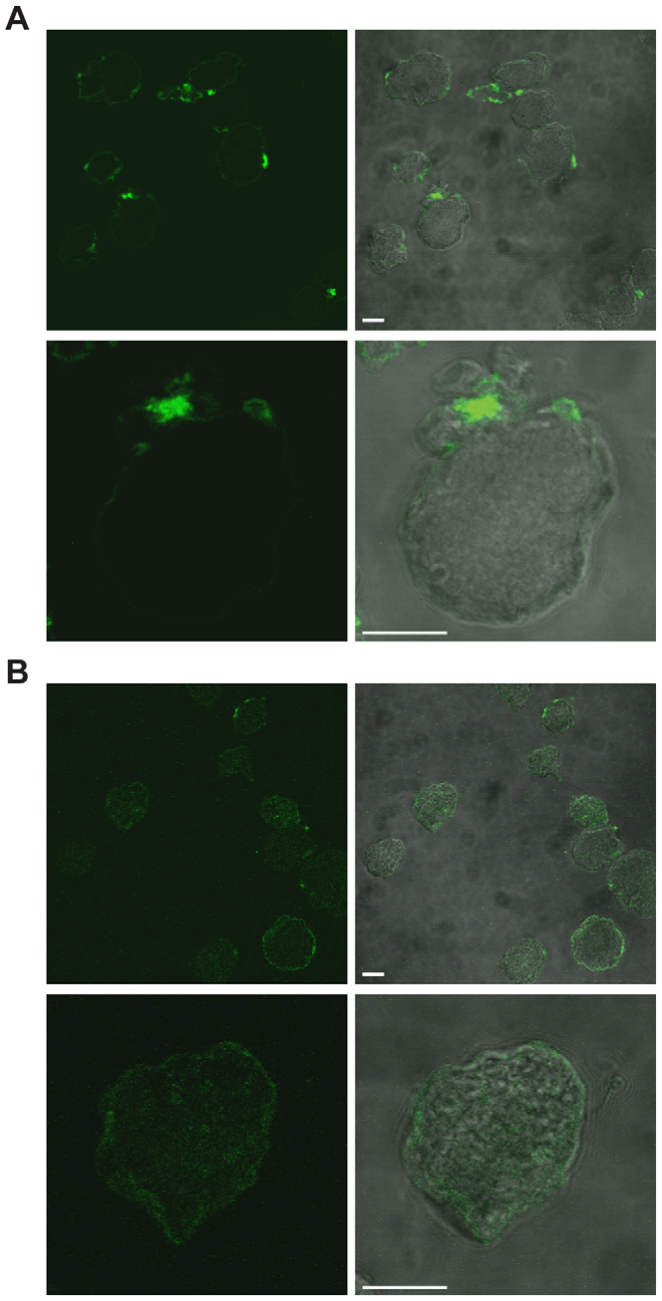
Serum from amoebiasis patients promotes uropod formation in *E. histolytica*. The micrographs represent confocal microsopy sections of fixed parasites following incubation with serum from patients with amoebic liver abscesses (A) or from healthy donors (B). The left panels show immunofluorescence images obtained after incubation with anti-human antibodies. The right panels show the overlaid phase contrast/fluorescence images of entire parasites. The detector gain for fluorescence was increased in images shown in panel B. Scale bars: 10 µm.

Previous work had suggested the release of membrane fractions from trophozoites after incubation with serum from patients suffering from hepatic amoebiasis [7]. We analyzed the ability of human serum (from both healthy individuals and *E. histolytica*-infected patients) to induce surface receptor capping and uropod formation. For each serum tested, parasites were fixed and the presence of caps was determined by epifluorescence using an anti-human secondary antibody (Figure 2). Sera from healthy individuals bound weakly to the surface of *E. histolytica*. Small membrane patches were seen in only 8% of cases (46 out of 582 amoebae, counted in three experiments). In contrast, cell binding and efficient uropod formation (52%: 282 out of 542 amoebae, counted in three experiments) was clearly observed when sera from patients presenting liver abscess were used.

### LC-MS/MS analysis of protein content in uropod extruded fractions

**Figure 3:**
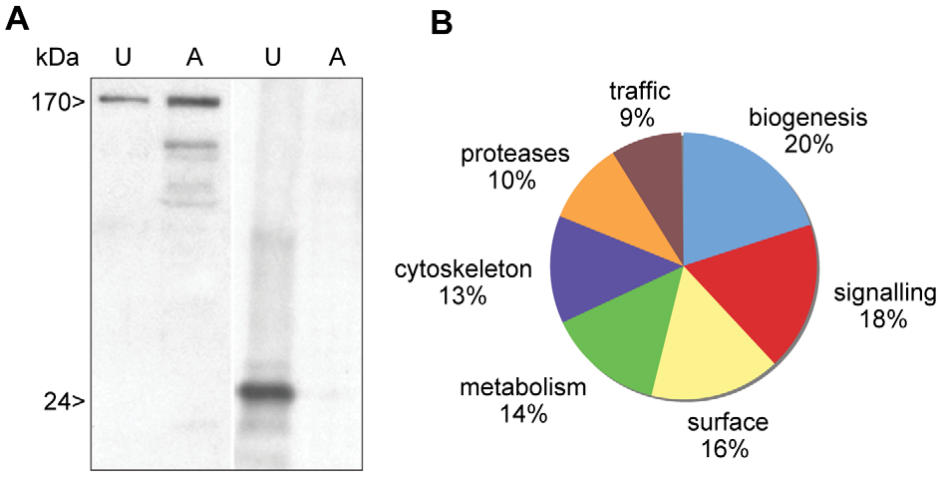
Distribution in functional categories of the proteins present in the *E. histolytica* uropod extruded fraction. A. Electrophoretic analysis of proteins from the ConA-uropod complex and from crude extract. A sample of UEF or amoebic extracts (10 µg, U = uropod; A = amoebae) were resolved by SDS-PAGE. The Gal/GalNAc lectin heavy chain (170 kDa) and the Con A (24 kDa) were revealed by western blot. B. Protein identification with LC-MS/MS was followed by proteome comparisons using the BLAST computer program, GO annotations and manual annotations. Two LC-MS/MS experiments were performed. Only proteins identified by at least two peptides in each experiment were taken into account. In all, 104 proteins were present in both experiments and could be analyzed. The entire data set was submitted to Tranche (https://proteomecommons.org/tranche/) database.

Uropod extruded fractions (UEF) were recovered, treated as indicated in methods section and submitted to western blot analysis in order to identify the heavy chain of the Gal/GalNAc lectin and Con A as a control (Figure 3A). We applied a high-throughput proteomics approach to uropod-extruded fractions and gained insight into the potential mechanism of surface receptor capping and the signal transduction pathways that induces cap formation and release. After capping induction in 10^8^ cells from two independent experiments, UEF were analyzed by LC-MS/MS and the peptide sequence data were determined (see Methods) with a Xcorr higher than 1.5 and 0,2% of percentage of false discovery (FDR). A list of proteins was generated taking into account for protein identification 99,9% accuracy, among these we analyzed proteins represented at minimum by two peptides. A set of 269 proteins was established (The entire data files were submitted to Tranche database (https://proteomecommons.org/tranche/). Whereas 36 of these were hypothetical proteins with unknown functions, 104 proteins were present in both experiments and could be categorized using both functional GO-term annotations and manual annotation via BLASTP and InterProScan (for protein domain searches) from the EMBL database (Figure 3B and Table S1). Signalling molecules accounted for a significant proportion of the UEF proteome, with the most numerous being small GTPases from the Rho and Rab families. Metabolic enzymes, biogenesis factors and trafficking-related molecules were present in the UEF proteome. These proteins are linked to plasma membrane and to the endocytic process. Surface molecules, cytoskeletal proteins and amoebic proteases were also identified. Lastly, a potential virulence factor (KRiP3) was found in UEF. We further characterized the surface proteins, the cytoskeleton proteins and the proteinases since these categories are potentially involved in the surface receptor capping process and the anti-amoeba immune reactions (Table 1). In addition to the stringency of protein selection, one important criteria allowing us to goes further in this analysis was the fact that for example surface antigens such ARIEL [40], kinase receptors abundant family [41] or β-tubulin (nucleus marker) were not present in this proteomic analysis indicating that we have in the analyzed fraction proteins mostly linked to UEF.

**Table 1:** Surface-linked proteins in the uropod extruded fractions.

				Experiment I		Experiment II	
Genbank GI	JCVI Accession	Description	Mass (kDa)	Unique peptides	% Covery	Unique peptides	% Covery
67479719	EHI_136160	Calreticulin	45	12	29	11	32
67478183	EHI_015380	Immuno-dominant variable surface antigen M17	125	11	10	8	11
405076	N/A	P-glycoprotein 6	143	8	6,9	10	8
183232088	EHI_100320	Multidrug resistance protein	182	8	6,6	9	6,8
183232225	EHI_030830	Plasma membrane calcium-transporting ATPase	114	8	8,5	8	9,2
67475672	EHI_016480	Plasma membrane calcium-transporting ATPase	119	8	4,8	5	4
3392885	N/A	Plasma membrane calcium -transporting ATPase	121	7	6,3	3	3,6
67481663	EHI_012270	Gal/GalNAc lectin heavy subunit	144	6	4,8	7	5
67481591	EHI_035690	Gal/GalNAc lectin light subunit	34	5	2	4	20
67476079	EHI_065670	Cation-transporting P-typeATPase	126	5	2,7	3	3,1
67484480	EHI_148790	Gal/GalNAc lectin light subunit	32	4	17	5	13
305078	N/A	Gal/GalNAc lectin light subunit	34	4	12	2	6
183230108	EHI_012330	Serine-Threonine-Isoleucine Rich Protein	291	4	1,8	2	1
67475812	EHI_074020	Vacuolar proton ATPase subunit	93	3	5,5	7	12
67463605	EHI_111990	CXXC-rich protein	131	3	2	4	3
67474486	EHI_181220	Adhesin 112 (EhADH112)	78	3	5,7	3	8,2
67479029	EHI_095820	ATP-binding cassette transporter MRP	152	3	2,3	3	2,4

### Amoebic proteins associated with the cell surface and present in the uropod extruded fraction proteome

The Gal/GalNAc lectin protein complex (within which HgL and LgL subunits were identified) and CRT were representative of amoebic surface-related protein as expected (Table 1). The presence of the lectin complex in caps has been observed by the use of a range of molecular and cellular methods [11], [15], [42]; as well as the presence of CRT [24]. One important surface protein found at the UEF was the 125 kDa immunodominant antigen M17, which is recognized by sera from patients with amoebic liver abscesses [35]. In addition, the surface related proteins (EHI_100320, EHI_030830, EHI_016480 and EHI_074020) contain an ATP-binding cassette from the ABC transporters superfamily. Pgp6 (EHI_101230) is constitutively expressed in parasites which are resistant to the anti-amoebic drug emetine and is involved in the multiple drug resistance phenotype [36]. We also identified the adhesin ADH112 (EHI_181220), which is part of a surface and vacuolar heterodimer complex involved in adhesion, cytopathic processes and phagocytosis [43], [44]. ADH112 has a cell adhesion domain at its carboxyl terminal and a Bro-1 signalling domain at its amino terminal [45]. Interestingly, ADH112 shows homology with Alix, a factor that regulates integrin-mediated cell adhesions and extracellular matrix assembly [46]. Another parasite adhesion protein found in the UEF was the serine-, threonine- and isoleucine-rich protein (STIRP). The latter is predicted to be a transmembrane protein encoded by a five member multigene family. It is only present in pathogenic *E. histolytica* [47] and its inactivation reduces parasite adhesion to cultured epithelial cells. Our data indicate that STIRP is a membrane associated component. Lastly, an unknown protein from the CXXC motif-containing family was present. It has a signal peptide and seven furin-like cysteine rich regions that is found in a variety of proteins and involved in signal transduction via receptors tyrosine kinase [48].

### Cytoskeleton-related components present in the uropod extruded fraction proteome

Several proteins linked to the actin-rich cytoskeleton were identified in the UEF proteome (Table 2). Most have already been observed in the uropod region during capping and include actin [15], myosin II heavy chain [13], [26], the small GTPase Rac G [27], guanine exchange factors [29], filamin [34] and α-actinin [19]. Moreover, our proteomic analysis highlighted the signalling pathway leading to surface receptor capping through the discovery of filopodin (EHI_167130) - an uncharacterized protein with three ezrin/radix/moesin (ERM) domain repeats and one I/LWEQ domain (which binds to actin and is present in talin). Talin has an important role in the interaction between the cytoskeleton and the cell surface receptors [49] and also influences ERM protein function during uropod induction in T lymphocytes [50]. For instance, this is the first report to identify ERM domain-containing protein (which is pivotal for capping of adhesion molecules in lymphocytes) in an evolutionary early branching eukaryote such as *E. histolytica*. This finding suggests an ancient origin for the ERM domain and opens up opportunities for further molecular studies on cytoskeletal activities during receptor capping in *E. histolytica*. The UEF proteome analysis revealed that several actin-binding proteins are related to the spectrin-like protein family (e.g. α-actinin and filamin). These proteins have already been identified in the *E. histolytica* uropod using cell biology techniques [19], [34]. Spectrin family proteins and the associated kinases are known to redistribute to the uropod following T cell activation during the onset of inflammation [51]. The dynamics of actin filaments within the uropod was also illustrated by the presence of factors such as the p41-Arc component of the Arp2/3 complex that is involved in “de novo” actin filament formation [52]. Calcium is one of the most versatile and universal second messengers in cells. It is widely accepted that intracellular Calcium has an effect on the actin cytoskeleton dynamics. Although the calcium-binding proteins of unknown function grainin 1 and grainin 2 were highly abundant in the UEF, a functional link between grainins and the cytoskeleton has not yet been reported in the literature.

**Table 2:** Cytoskeleton-related and proteinases in UEF.

				Experiment I		Experiment II	
Genbank GI	JCVI Acession	Description	Mass (kDa)	Unique peptides	% Covery	Unique peptides	% Covery
**CYTOSKELETON**							
67483616	EHI_110180	Myosin II heavy chain	247	26	14	40	22
67462785	EHI_159150	Actin	42	10	28	14	38
67468658	EHI_167310	Grainin 2	24	7	22	15	38
67468717	EHI_167300	Grainin 1	24	5	25	8	33
183230870	EHI_155530	Chromosome partition protein	121	3	1,9	2	1,6
103484580	N/A	Clathrin heavy chain	184	2	4,8	8	5
6636336	N/A	Actinin-like protein	63	2	4,8	5	9,7
67478790	EHI_167130	Filopodin	180	2	1,5	5	3,3
67484080	EHI_045000	Actin-related 2/3 complex subunit 1A	40	2	6,6	4	15
67484714	EHI_148890	Calmodulin	17	2	14	4	29
183234431	EHI_120360	Grainin	25	2	12	3	16
67484090	EHI_104630	Filamin 2	95	2	3,5	2	3,2
67477667	EHI_110810	Unconventional myosin IB	119	2	2	2	2,7
**PROTEINASES**							
183231030	EHI_127030	Peptidase-CP-C6	58	7	12	5	14
67469327	EHI_033710	Cysteine proteinase, CP-A2	35	4	15	6	18
67479681	EHI_136440	Dipeptidyl-peptidase (lipase family)	77	4	4,9	5	15
183231521	EHI_093970	Peptidase-CP-C13	69	4	6,6	3	7,9
67463512	EHI_182720	Dipeptidyl-peptidase ((lipase family)	76	3	6,5	7	11
183231582	EHI_010340	Peptidase-CP-C5	64	3	5,4	3	5,6
67465637	EHI_037190	Serine carboxypeptidase Sp2	54	2	2,7	5	5,2
544088	EHI_074180	Cysteine proteinase, CP-A1	35	2	5,6	4	14
67480901	EHI_152220	Peptidase-CP-C4	58	2	4,6	3	7,4
67469932	EHI_168240	Cysteine proteinase, CP-A5	35	1	5,3	3	7,9

### Amoebic proteinases present in the UEF proteome

Proteases were another category of the main factors found in the UEF (Table 2). The cysteine proteases were all endopeptidases (seven in total): CP-A1, CP-A2 and CP-A5 from the very well known A family and CP-C4, C5, -C6 and -C13 from the C family. The C family was recently discovered in *E. histolytica* [53]. Several studies have shown that peptidases (particularly cysteine peptidases) are major pathogenicity factors in *E. histolytica* [54]. CP-A5 is the prime candidate, (although we only found one peptide in experiment I which however covers 5% of the protein), since it localizes at the amoebic surface [55] and is involved in human colon invasion [18] and ALA formation [56]. This protease contains an Arg-Gly-Asp (RGD) integrin binding motif which has also been found in the proregion of cathepsin X from higher eukaryotes [57]. In cell-adhesion proteins like fibronectin, RGD motifs serve as ligand recognition sites for cell-surface receptors such as the integrins. Recently, it has been shown that the RGD motif present in the pro-form of amoebic CP-A5 binds to the integrins of intestinal Caco2 cells and promotes the activation of the NFκB signalling pathway [38]. In addition to cysteine proteinases, we also identified Sp2 one of the members of a family of three amoebic serine proteases (i.e. S28 family) [58] and two dipeptidyl-peptidases from the lipase family which hydrolyze tryglycerides, phospholipids and cholesterol esters [59].

In summary, the present article reports the main features of the proteomic profile obtained by LC-MS/MS analysis of *E. histolytica* uropod fractions. The released surface proteins, cytoskeleton-related proteins and cysteine proteases identified herein might help us to understand the mechanism of surface receptor capping and uropod formation. Given that the Gal/GalNAc lectin complex is widely described as being involved in capping [13], [14], [15], we decided to extend our proteomic analysis by studying the cysteine proteases' roles and M17's localization during uropod formation.

### Inhibition of cysteine proteinases does not change surface receptor capping and uropod formation

**Figure 4:**
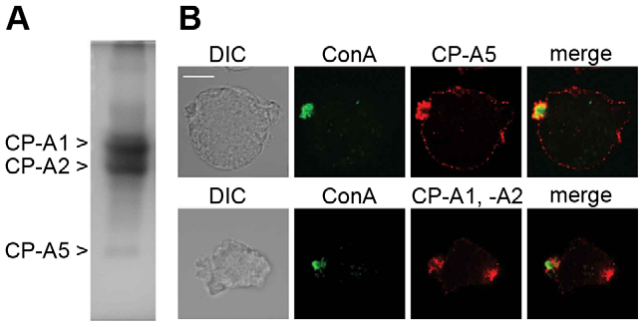
Cysteine proteinases are present as pro-enzymes and active enzymes in the uropod extruded fractions. A. Substrate gel electrophoresis of uropod extruded fractions (1 µg of proteins), which were separated by electrophoresis in SDS-PAGE co-polymerized with gelatine. To visualize the cysteine proteinase activity, gels were stained with Coomassie blue. The figure shows the inverted image. B. Cellular localisation of CP-A5, -A1 and -A2 in *E. histolytica*. Trophozoites were incubated with Con A (green). Upon incubation, the cells were fixed and stained for CP-A5 (up panel) or CP-A1 and -A2 (low panel) with specific antibodies (red). Scale bar: 10 µm.

Active cysteine proteinases (such as cathepsin X) have been shown to interact with β2 integrin and to cause cytoskeletal rearrangements that stimulate T lymphocyte migration and uropod formation. Membrane-associated *E. histolytica* cysteine and serine proteases may have a role in the degradation of the tight junctions of target cells, since it has been reported that use of the corresponding inhibitors prevents this process [58], [60]. Therefore, we sought to investigate whether or not cysteine proteases present in the UEF have hydrolytic activity and so determined the protease activity of this fraction in a gelatin gel assay (Figure 4A). The data evidenced a good correlation between the patterns generated by peptidases present in the UEF on one hand and the digestion pattern previously published carrying CP-A1, CP-A2 and CP-A5 activities on the other [61]. The data also corroborated the previous report in which 48, 34 and 17 kDa bands are associated with proteolytic activity and corresponded to CP-A1, CP-A2, and CP-A5, respectively [61]. We thus can infer that active cysteine proteinases were present in the UEF. We confirmed by immunofluorecence the presence of CP-A1, -A2 and -A5 on the uropod of trophozoites incubated with Con A (figure 4C). Interestingly, in addition of the uropod, the antibody detecting both CP-A1 and -A2 stained also at the leading edge of *E. histolytica*, whereas the CP-A5 stained more accurately the membrane surface. In order to investigate the impact of cysteine proteinases in *E. histolytica* uropod formation, we determined the influence of cysteine protease inhibitors on the uropod formation efficiency. Live parasites were incubated in the presence of either cell-permeant E64 (which acts on both extra and intracellular CPs) or cell-impermeant E64 (which acts on extracellular CPs only) at 100 µM. The number of uropod-positive cells was not significantly lower in the presence of these inhibitors in three experiments performed. To investigate whether CP-A5 has a specific role in uropod formation, the behaviour of *E. histolytica* silenced for CP-A5 gene expression [20] (i.e. RB8 strain) was examined. Incubation of RB8 parasites and its parental strain G3 with Con A showed that the parasites had equivalent uropod formation rates (two experiments performed). This finding indicated that although cysteine proteinases are abundant and active in the uropod fraction, they do not influence the dynamics of receptor capping or uropod formation. Cysteine proteinases are important for pathogenicity in *E. histolytica*; given their abundance in the extruded amoebic uropod fractions, we expected them to have much the same functions in surface receptor capping and uropod formation as they do in leukocytes. However, inhibition of cysteine protease activity did not significantly modify the efficiency of cap formation and thus emphasized a contrast with the known role of cysteine peptidases in leukocyte uropod formation.

### Serum from amoebiasis patients induces M17 enrichment at the uropod

**Figure 5:**
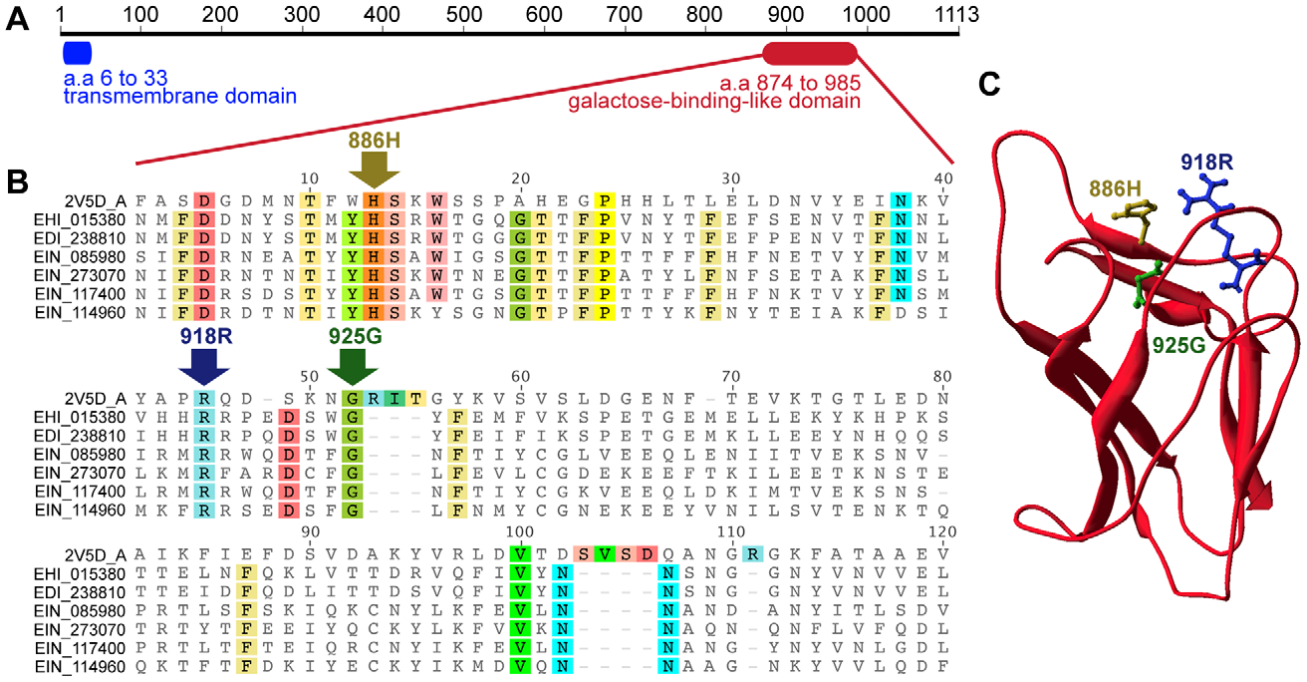
Conservation of carbohydrate-binding residues in the galactose-binding-like domain of M17 homologues of *Entamoeba*. A. The domain architecture of M17 (EHI_015380). The transmembrane domain and the galactose binding-like domain (IPR008979) were identified using Philius [66] and InterProScan software packages, respectively. B. The amino acid sequence alignment of the carbohydrate-binding domain of M17 homologues in *Entamoeba* and GH84C of *C. perfringens* (PBD ID: 2V5D_A). Residues with >75% identity are highlighted. M17 homologues of *E. dispar* (prefix EDI) and *E. invadens* (prefix EIN) were identified using BLASTP analysis of their proteomes, with M17 as the query. The arrows indicate the carbohydrate binding residues in GH84C [62]. C. Predicted structural model of the galactose-binding like domain of M17. The model was predicted from the 3D jury meta-server [67], with *C. perfringens* GH84C as the best-hit template (i.e. the template with the highest 3D-jury score = 81.56; score of 50 is the default cut-off, which results in a prediction accuracy of above 90%). The side-chains of the three conserved carbohydrate binding residues are coloured and labelled as in panel B.

Except for the detailed results on the Gal/GalNAc lectin's capping at the amoebic surface and localization of CRT in the uropod, there are no literatures describing other capped surface molecules in *E. histolytica*. One of the major goals of the present work was to identify surface molecules that might have an important role in the development of amoebiasis and/or the onset of immune responses against *E. histolytica*. These objectives prompted us to analyse further M17 in the process of surface receptor capping. We first investigated the domain architecture of M17. This protein was predicted to contain an N-terminal transmembrane domain and a galactose-binding-like domain (Figure 5A). Galactose-binding-like domains (InterPro: IPR008979) are structurally conserved as a beta-sandwich and are responsible for binding to specific ligands, such as cell-surface-attached carbohydrate substrates and phospholipids on the outer face of the mammalian cell membrane. In fact, meta-prediction of the structure of M17's galactose-binding-like domain suggested that its three-dimensional folding is similar to that seen in a number of prokaryotic carbohydrate binding proteins. Indeed, the best hit of this meta-prediction was an extracellular carbohydrate-active virulence factor from *Clostridium perfringens*, GH84C [62]. Sequence alignment of M17 homologues in *Entamoeba* species and GH84C suggests the conservation of three critical carbohydrate-binding residues (Figure 5). Hence, M17 is likely to be located on the cell surface and has a potential role in the carbohydrate-mediated binding of the amoeba to its host cells [63].

**Figure 6:**
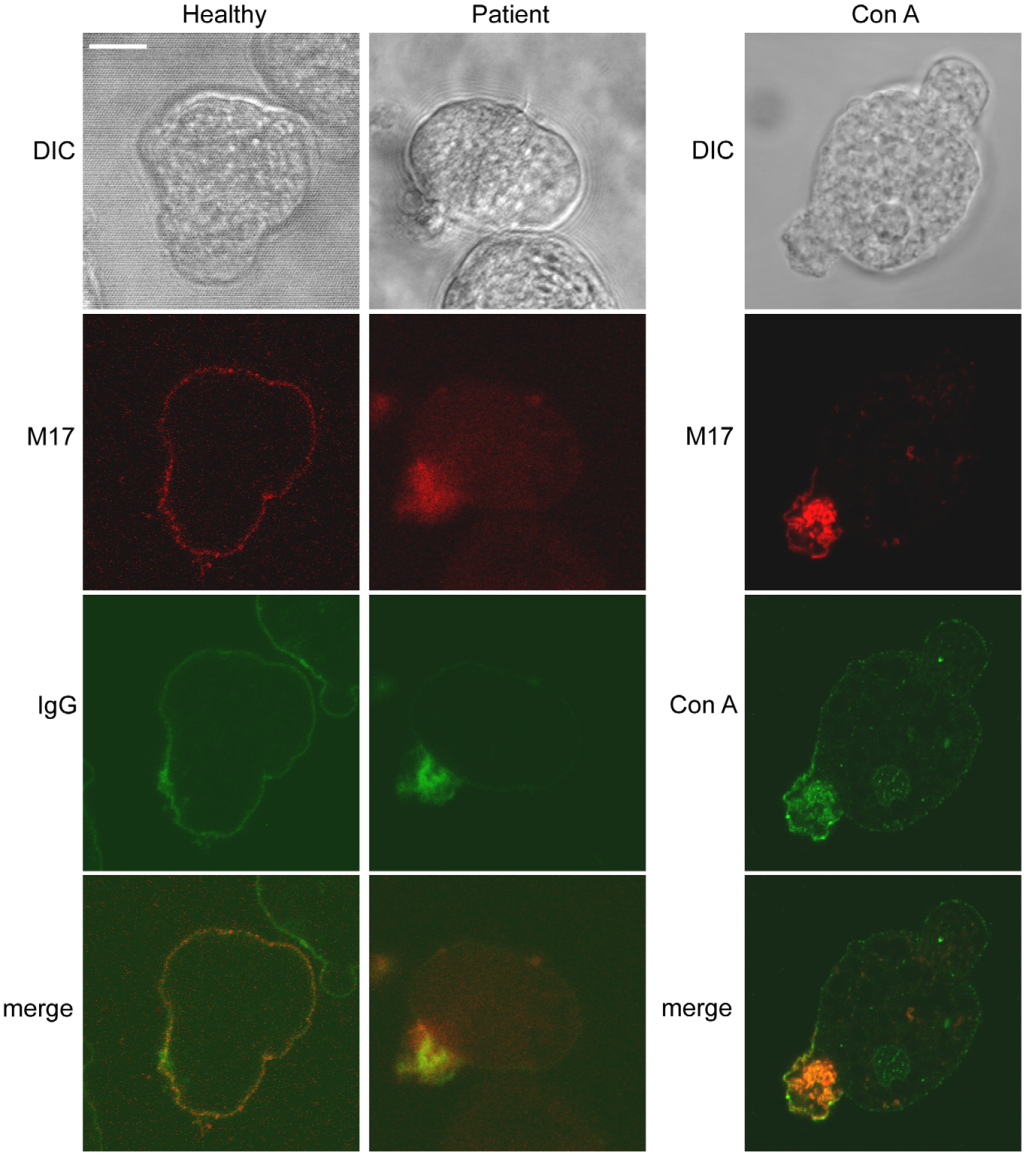
The cellular localization of the immunodominant antigen M17 in *E. histolytica*. Trophozoites were incubated with serum from healthy patients (left panels), with serum from patients with amoebic liver abscesses (middle panes) or with green fluorescent Con A (right panels). Upon incubation, the cells were fixed and stained for M17 with a specific antibody (red) and co-stained with a serum recognizing anti-human IgG (left and middle panels). Scale bar: 10 µm.

In previous work, antibody-antigen caps were induced by incubation of *E. histolytica* with an anti-M17 monoclonal antibody [35]. However, given the absence of control experiments using unrelated monoclonal antibodies in the initial report, cap formation may have been caused by the mere presence of immunoglobulins (regardless of their specificity) in the antibody fraction. The abundance of M17 in the UEF proteome and its potential role in amoebic physiology prompted us to perform a cellular analysis of M17 during the receptor capping process. We generated a specific anti-M17 antibody for use in western blots and for immunolocalization studies in entire cells. Confocal microscopy analysis clearly demonstrated that M17 localized to the amoebic plasma membrane (Figure 6). To determine the relevance of M17 translocation to the uropod, we looked at whether this protein appeared at the uropod following cell activation with Con A and following incubation of *E. histolytica* with serum from patients with amoebiasis. Staining with the specific anti-M17 antibody and high-resolution confocal microscopy revealed that in both instances, M17 translocated to the rear cell region (Figure 6). Furthermore, at least half of the uropods formed after exposure to sera from patients contained the M17 (two sera were tested). These results clearly showed that M17 is not only on the parasite surface but is also recruited to the uropod following incubation with serum from amoebiasis-positive patients (and not naive sera). The complexity of the uropod protein fraction being discarded to the external medium raises the question for further exploration of the interplay between circulating M17 and other immunodominant antigens in amoebiasis-triggered immune responses.

## Discussion

The uropod is a dynamic structure generated at the rear of polarized mobile cells. It trails the cells and contains various surface proteins. Depending on the nature of the capped surface proteins, a broad panel of biological functions can be associated with this structure. Uropods might be functionally involved in (i) connecting intercellular stalks which facilitate cell-cell interactions in processes such as antigen transport, cytotoxicity, leukocyte extravasation and apoptosis, (ii) providing mechanical forces necessary for motility and cell deformability by facilitating cell passage through constricted spaces and (iii) serving as a site of active bidirectional traffic, in which endocytosis and exocytosis are regulated in a coordinated manner [3]. Furthermore, it has been suggested that *E. histolytica* uses uropods to escape from the host's immune responses; it has been shown that during actomyosin II-based contraction, uropods are released into the external medium and lead to an accumulation of ligand-receptor complexes. In some cases, the ligands (such as antibodies and complement) have anti-amoebic activities. The discarded fraction might also have a role in triggering further steps in the immune response during parasite invasion. Our study showed that sera from amoebiasis patients (but not naïve sera) induced a remarkably clustering of molecules in the uropod of *E. histolytica*, suggesting that surface molecule clustering may have a significant impact on the immune response. This finding is critical for future developments in diagnosis and/or vaccination against *E. histolytica*, since the molecules discarded through uropod release circulate in the blood and are very likely to enter into contact with endothelial cells and with immune cells in charge of molecule clearance, antigen presentation and the induction of inflammation.

Here, we have reported that stimulation of *E. histolytica* with the lectin Con A as an experimental model enabled the analysis of the initial capping, then uropod formation and the dynamics of capping in living cells. The engagement of ligand-receptor interactions at the amoebic surface prompted a very rapid change in cell morphology, followed by uropod formation and the extrusion of membrane fractions. We identified *E. histolytica*'s uropod-associated proteins by performing a proteomic analysis of the released fractions. We determined the presence of disease-relevant surface molecules which are important candidates for the interaction of *E. histolytica* with human cells, including the Gal/GalNAc lectin, CRT, STIRP and ADH112 proteins. Furthermore, we described the clustering of the immunodominant variable antigen M17 - an abundant component of caps formed using either Con A or serum from infected patients. The latter findings indicate that this protein is important for eliciting an immune response. However, genome sequencing has shown that M17-encoding genes also exist in various non-pathogenic *Entamoeba* species (Figure 5). Additional studies will be needed to determine the special features of this antigen in pathogenic species.

We also identified molecules known to regulate actin-based cytoskeleton activities; this revealed a clear difference between uropods in amoebae and those in immune cells. For example, leukocyte uropods contain a microtubule (MT) organizing center. However, the fact that MT disruption in leukocytes does not impair uropod formation suggests that MTs (which are nuclear in *E. histolytica*) are not essential for this process. In contrast, cell polarization and uropod formation in *E. histolytica* are mainly regulated by polymerized actin networks maintained by spectrin-family actin-binding proteins. The latter include α-actinin and the filamins, which were previously found to accumulate at the uropod and interact with the COOH-terminal domain of the Gal/GalNAc lectin [14]. Our proteomic analysis newly identified an ERM-domain containing protein. In cells, ERM proteins act as membrane–cytoskeleton linkers by interacting with the amino-terminal domains of membrane proteins and the carboxyl-terminal domain of F-actin [64]. The proteins are pivotal in the signal transduction pathway triggered by receptor capping. For instance, it has been shown [50] that the preferential localization of ezrin (an ERM containing protein) in the uropod of leukocytes requires Thr567 phosphorylation and induces enhancement of uropod integrity, chemotaxis and polar cap formation. Interestingly, some transmembrane adhesion molecules (including CD43, CD44, intercellular adhesion molecules, and PSGL-1) are concentrated at the uropod in immune cells [3] because they have a motif within the intracellular domain which can bind to ERM-containing proteins.

A striking difference between uropods from human cells and those in *E. histolytica* concerns the role of cysteine proteases. In migrating lymphocytes, cathepsin X localizes at the uropod and causes cytoskeletal rearrangements by modulating the activity of β2-integrin containing receptor LFA-1. The pro-form of cathepsin X carries a RGD motif (also present in CP-A5 from *E. histolytica*) which interacts with the integrin. The protease then cleaves the four last amino acids of the β2-chain, resulting in its binding to talin - a crucial step in uropod elongation and cell polarization [23]. In contrast, CP-A5 (as well as other CPs) does not have any activity in uropod formation - at least judging by the data obtained with protease inhibitors and the CP-A5-silenced strain. Alternatively, other proteases may have a role in uropod formation, despite the fact that knockdown of the rhomboid serine protease (which specifically localizes at the base of the cap, rather than in the cap itself) had no significant impact on cap formation [65]. The fact that cysteine proteinases from the C family were highly represented in the UEF make these factors relevant for further analysis. Although cysteine proteases has been found into internal vesicles and/or on the amoebic surface, little is known about the potential association of trafficking vesicles and uropod membranes and/or subcortical cytoskeleton, but we cannot exclude this possibility. For instance, the pseudopod at the front of the cells is devoid of vesicles. At the moment, we can not confirm these CPs are interacting with the cytoskeleton in *E. histolytica* but it is totally possible, since in leukocytes cathepsin X interacts with the tail of β-integrin in the cytosol upon activation by cathepsin B in the lysosomes [22]. Hopeful we can get more insight in this point when single cell analysis will be performed trying to determine the dynamics of vesicle traffic in *E. histolytica*.

In conclusion, *E. histolytica*'s trailing edge accumulates important molecules (such as adhesion receptors, immune response activators, cytoskeleton components and proteinases) following the activation of surface receptor capping. In human infection, extrusion of these molecules into the interstitial cell space or the blood can trigger immune responses against *E. histolytica*. These proteins are potentially powerful markers for (i) studying the mechanism underlying uropod formation; (ii) addressing the question of how their activity (or their presence) elicits an immune response and induces cell death when in contact with human cells.

## Supporting Information

Table S1 Proteins identified at the uropod of *Entamoeba histolytica*.
(0.05 MB XLS)

Video S1 Uropod formation in *Entamoeba histolytica*. Trophozoites were seeded on glass bottom culture dishes (MatTeck) and incubated at 37°C in the presence of 5 µg/ml of fluorescent Con A (Alexa fluor 488, Molecular Probes). Live parasites undergoing capping were imaged using a confocal microscope (40× objective) with a Nipkow disk device (Perkin Elmer). Images (10 per second) in a focal plane show fluorescence changes at the amoebic membrane reflecting the surface receptor capping and uropod formation phenomena.
(9.08 MB AVI)
